# An economic-research-based approach to calculate community health-staffing requirements in Xicheng District, Beijing

**DOI:** 10.1186/s12960-016-0152-5

**Published:** 2016-12-07

**Authors:** Delu Yin, Tao Yin, Huiming Yang, Qianqian Xin, Lihong Wang, Ninyan Li, Xiaoyan Ding, Bowen Chen

**Affiliations:** 1 Capital Institute of Pediatrics, 2 YaBao Road, #328, ChaoYang District, Beijing, 100020 China; 2 Community Health Association of China, Chaowai Street. 18 A-2603, ChaoYang District, Beijing, 100020 China; 3 Center for Community Health Service Management in Xicheng District of Beijing, 8 SanYi DongLi, Xicheng District, Beijing, 100055 China

**Keywords:** Health worker, Community health service, Health professional, Economic-research-based approach, Workload

## Abstract

**Background:**

A shortage of community health professionals has been a crucial issue hindering the development of CHS. Various methods have been established to calculate health workforce requirements. This study aimed to use an economic-research-based approach to calculate the number of community health professionals required to provide community health services in the Xicheng District of Beijing and then assess current staffing levels against this ideal.

**Methods:**

Using questionnaires, we collected relevant data from 14 community health centers in the Xicheng District, including resident population, number of different health services provided, and service volumes. Through 36 interviews with family doctors, nurses, and public health workers, and six focus groups, we were able to calculate the person-time (equivalent value) required for each community health service. Field observations were conducted to verify the duration.

**Results:**

In the 14 community health centers in Xicheng District, 1752 health workers were found in our four categories, serving a population of 1.278 million. Total demand for the community health service outstripped supply for doctors, nurses, and public health workers, but not other professionals. The method suggested that to properly serve the study population an additional 64 family doctors, 40 nurses, and 753 public health workers would be required.

**Conclusions:**

Our calculations indicate that significant numbers of new health professionals are required to deliver community health services. We established time standards in minutes (equivalent value) for each community health service activity, which could be applied elsewhere in China by government planners and civil society advocates.

## Background

China once had an excellent primary healthcare system that was a model for other countries; however, after market reforms in 1978, there was a shift in funding, from community health services (CHS) to specialized hospital-based care, with a mandate for health institutions to generate a large portion of their operating revenue. This led to a proliferation of specialists and the excessive use of drugs and high-technology diagnostic tests [1]. Since 1997, China has been committed to reshaping the system of urban CHS. The reforms aimed to position CHS as the foundation and entry point of the health system, supported by new policies and increased government investment. In 1999, 2006, and 2009, the central government issued instructions for the development of CHS in urban areas to support policies and actions [2–5]. Over the last 16 years, great strides have been made. By the end of 2013, the total number of CHS facilities had reached 33 965 nationwide, with 8488 community health centers (CHCs) and 25 477 community health stations [6], basically achieving the objective of “every street has at least one community health center” [7], and the number of community health professionals was 0.42 per 1000 residents, 0.08 more than in 2005 [6].

Yet, health human resources and population need has slowed the growth of CHCs. A shortage of community health professionals has been a crucial issue hindering the development of CHS [8, 9]. In 2006, the central government issued a standard for configuring health staff in urban communities to respond to the dilemma, which, to some extent, promoted the creation of community health professionals [10]. According to the standard, each CHC will configure two to three family doctors, one public health worker for each 10 000 head of population, and the ratio of doctors and nurses should reach 1:1, while the proportion of the other professionals accounts for less than 5% of the total [10]. In recent years, CHS have been further recognized and used by local residents, and the workload of community health professionals has become much higher than that of specialists in hospitals. In 2013, family doctors routinely saw 15.7 outpatients each day, while hospital doctors saw an average of 7.3 [6]. Moreover, in 2009, the Chinese government issued guidance on a National Essential Public Health Services Package (NEPHSP), aiming to provide similar public health services regardless of geographical area, and to expand the coverage of essential public health programs to all residents of China [11]. The NEPHSP greatly increased the workload of community health professionals. With the increased requirements on community public health services and the loss of community health professionals as a result of ineffective incentive mechanisms [12], demand for CHS could no longer be met, and the situation has steadily worsened. This has already affected the performance of the CHS system and hindered its crucial role as the gatekeeper of the health system [13]. An evaluation on programs regarding the community-based management of hypertension and type 2 diabetes mellitus patients in eight provinces showed the standardized management rates of patients with hypertension/type 2 diabetes mellitus patients were only 61.1 and 59.0% in 2014 [14]. Community health professionals need to work overtime for 1.5–2 h. The professionals’ satisfaction with income and workload were lowest, respectively 45.23 and 45.65% [15]. While there has been guidance provided about the ideal configuration of community health staff in urban communities, a decade ago, there has been little done more recently to understand the health human resource requirements for CHCs given the NEPHSP.

### Measuring workforce requirements

Various methods have been established to calculate health workforce requirements. Many use practitioner-to-population ratios, or historical patterns [16, 17], although there are other methods including case-load profiling, acuity measures, queuing theory, and production function [18–21]. In 1998, the World Health Organization (WHO) developed the Workload Indicators of Staffing Need (WISN) to calculate optimal allocations and use of staff [22–24]. Compared with the other methods, its results are relatively reasonable and accurate, which is helpful for healthcare workforce planning [23]. The method has previously been used in Tanzania, Namibia, and elsewhere. The problem with the WISN approach is the static and narrowly focused model underlying the WHO approach [20, 25].

### Purpose of this research

The purpose of this project was to use an economic-research-based approach to calculate the demand and supply of community health professionals in Xicheng District and to measure the staffing gap between the ideal number and current staffing levels. This provided a reference point and effective basis for assessing community health-staffing issues and formulating relevant policies in other areas in China and elsewhere.

## Methods

### Study setting

The study focused on the Xicheng District in Beijing City, the capital of China. The district is located in downtown Beijing and has 15 streets of offices and 255 community neighborhood committees. In 2013, the population in Xicheng District was 16 200 000, with a GDP of about USD 40.3 billion. In total, there are 620 health facilities in the district, including 15 tertiary hospitals, 14 secondary hospitals, and 78 community health stations affiliated to 15 CHCs. Currently, there are 2035 personnel working in community health facilities, of which 82.10% are community health professionals.

### Data collection

We focused our study on all types of services provided to the residents and restricted community health professionals to family doctors, nurses, public health workers, and other professionals including pharmacists and laboratory technicians. The study was further limited to 14 government-owned CHCs and their affiliated community health stations. The remaining CHC was hospital-owned and refused to provide data for the study. To ensure the on-site surveys and data collection could be done smoothly, a six-member research team was set up, including the health administration officials of Xicheng District, experts in healthcare human resources, agency officials, doctors, nurses, and public health physicians, supported by the local health administration department. Oral consent was provided by the health professionals and agency heads before their participation in any discussions or interviews.

Data for the calculation were collected as part of a larger study from 14 CHCs in Xicheng District in 2013. A standardized form was used to collect basic information, including the resident population, current number of community health professionals, and volume of each type of CHS. CHC managers were trained on how to collect the necessary data. Data were collected from the CHS information system over a 2-week period. Two separate researchers conducted site visits to oversee the data collection.

### Demand calculations

A report on the performance evaluation of CHS nationwide conducted in 2011–2013 showed that the quality of some CHS was lower than required in the guidelines because of a shortage of community health professionals [26]. We calculated the total demand for CHS that would be generated if all the services were delivered at the level of quality set out in the medical, nursing, and public health guidelines. We then estimated the total workload indicator (person-time) required to provide all the CHS for the study population for the period from January 1 to December 31, 2013. Demand was also measured based on the actual volume of CHS used by the residents in 2013 in the sample areas.

### What components are included in community health services?

China’s community health facilities were designed to deliver comprehensive primary healthcare services, from basic medication to rehabilitation, and public health services including the NEPHSP, which covers health education, children’s health services and immunization, maternal health services, older people’s health management, and services for patients with chronic diseases [27, 28]. According to research conducted in 2014, public health work accounts for about 45% of the total workload in urban community health facilities nationwide [29]. To calculate demand, we catalogued all these types of CHS and classified them into five categories by staffing requirements: essential medical services, nursing, pharmacy, auxiliary examinations, and public health services.

### How much time does it take to deliver each service unit?

We estimated the workload indicator for each service unit based on two steps: (1) determining the standard service protocols of all types of CHS and (2) defining the workload indicator for a set of standard activities for each service, and their equivalent value (EV) compared with a standard clinic visit. This method has been used to estimate the cost of the National Essential Public Health Services Package in Beijing, China [30].

### Step 1: determining the standard service protocols

All types of CHS provided by the sampled CHCs were investigated. In total, 134 types of CHS were deemed necessary for inclusion in the standard service protocols, including medical (35), nursing (15), paramedical (5), public health services (65), and auxiliary examinations (14). The main types are listed in Table 1. Only 10 of the 134 were included in the 2011 NEPHSP guidelines [31]. The remaining 124 types of CHS were defined in line with the 2007 Beijing technical specification for CHS [32].

**Table 1:** Workload and EV of the main CHS compared with a standard clinic visit

Categories	Types	Workload (minutes)	Mean EV
Essential medical services	Single clinic visit	15.00	1.00
	Emergency (per visit)	37.50	2.50
	Single home visit	60.00	4.00
	Inpatient bed day	90.00	6.00
	Rehabilitation clinic (per outpatient visit)	40.00	2.67
Nursing services	Intravenous injection	5.00	0.33
	Intravenous infusion	12.00	0.80
	Intravenous injection, venous blood	7.00	0.47
	Catheterization	20.00	1.33
	Providing prescription (Western medicine)	3.00	0.20
Pharmacy service	Dispensing a prescription (per prescription)	12.50	0.83
	Advanced pharmacy work, including detailed dosage calculations (per prescription)	75.00	5.00
Auxiliary examination service	Rapid blood sugar test	3.00	0.20
	Blood, urine, feces test (per test)	13.00	0.87
	Biochemical test (per test)	30.00	2.00
	Electrocardiogram (per test)	5.00	0.33
	B-mode ultrasonography (per test)	15.00	1.00
Public health service	Health record management service (per person year)	50.00	3.33
	Health education service (per month)	1400.00	93.33
	Health services for children aged 0 to 36 months (per person year)	106.67	7.11
	Maternal health services (per person year)	296.00	19.73
	Older people’s health services (per person year)	70.00	4.67
	Immunizations (per visit)	26.55	1.77
	Patients with hypertension (per person year)	310.00	20.67
	Patients with type II diabetes (per person year)	310.00	20.67
	Patients with severe mental illness (per person year)	510.00	34.00

### Step 2: calculating the workload indicator and the EV of each CHS

To create the workload indicator (person-time) for each CHS, a multi-stage iterative feedback and revision process was conducted. A series of four meetings were held with 42 invited participants with particular knowledge and expertise about CHS. Attendees included 14 CHS managers, 14 family physicians, 6 nurses, and 8 public health workers. During the meetings, participants discussed the staffing and time requirements for each CHS, the workload assigned to each in the standard service protocols, and possible modifications to this.

To test these workload indicators, 6 CHCs were randomly selected from the sample of 14 to participate in direct observations. Five research assistants were trained to observe the services and record the length of time for each CHS and the number and staff group of health workers involved. Direct observation took place over a period of three continuous days in each CHC. Face-to-face interviews were conducted to determine the usual time and the required number of health workers for each service, to check against the direct observations.

The workload indicators were modified based on the direct observation and interviews. Group interviews with 20 staff at the eight centers were conducted to test the workload indicators. Six family physicians, four nurses, seven public health workers, and three other health professionals participated in the interviews.

To ensure that the different types of CHS can be directly compared, a “standard clinic visit” was introduced as a benchmark to gauge the necessary staff and time required (workload indicator) for the other services. A standard clinic visit was defined as a family physician consulting with one patient for 15 min [33]. The workload indicator of a standard clinic visit was defined as one EV. EVs for all other CHS were then calculated by comparing their workload indicator from step 2.1 with the standard clinic visit. For example, a home visit may include the time taken to travel to and from the patient’s home and to administer medication. The workload indicator of one home visit was 60 min in both urban and suburban areas, so its EV was 4 (60 ÷ 15). The EV of each CHS is shown in Table 1.

### What is the total workload required to serve the study population, by staff group?

The volume of each service in 2013 was multiplied by the EV for each, and these figures were summed to find the total EVs for all 134 types of CHS across the sampled CHCs. This gave a figure for the total workload required to serve the study population. Nurses generally had broad responsibilities extending beyond traditional nursing roles, including involvement in the provision of basic medication and public health services. The group interview participants estimated that nurses spent 30% of their time on public health services.

### Supply calculations

We calculated the available supply of community health professionals in the 14 CHCs. We multiplied days worked by working hours per day and subtracted holidays, weekends, meal times, and rest time for each working day. Based on the interviews with community health professionals, each health professional generally works for 250 days in a year, for six effective working hours per day. The EVs per year of a full-time professional are therefore 6000 EVs (=(250 days × 6 h × 60 min) ÷ 15 min, because 15 min is the workload for one EV). We then used the following formula to calculate the supply:

$$ \mathrm{Supply}\kern0.5em =\kern0.5em {\displaystyle \sum \mathrm{Number}\ \mathrm{of}\ \mathrm{community}\ \mathrm{health}\ \mathrm{professionals}\ \mathrm{in}\ \mathrm{the}\ 14\ \mathrm{CHCs} \times 6000\ \mathrm{E}\mathrm{V}\mathrm{s}}. $$

### Gap and surplus calculations

We subtracted total demand EVs from total supply EVs to derive the gap or surplus EVs available for community health professionals in the 14 centers. We then divided these gap or surplus EVs by the number of EVs per year that a full-time professional would expect to work, to determine the equivalent number of full-time community health professionals required to fill the gap or in excess. We then calculated the ratio of total supply EVs to demand EVs to estimate the magnitude of the gap or surplus. A ratio of 1 implies perfect balance, below 1 indicates a gap and above 1 indicates a surplus.

## Results

### Staffing sample CHCs

Each sample CHC had an average of 125 employees on the payroll (1752 employees in 14 centers), serving an average community of 91 400 people (a total of 1.278 million people). The majority of community health professionals were doctors (49.6 people per center), followed by nursing staff (37.1), then public health workers (19.7). There were 18.6 other community health professionals per center, including pharmaceutical, imaging, and inspection personnel, but not logistics or administrative staff.

### Demand calculations

For the 14 CHCs in Xicheng District, the total demand in 2013 was about 17.18 million EVs. Public health services made up 51.3% of the total demand, which more than basic medical services (see Table 2). The average EVs for each center were 1 226 857.

**Table 2:** CHS demand by category of service across 14 sampled CHCs

Categories	Demand (EVs)	Percentage
Basic medical services	4 556 299.2	26.5
Nursing services	710 181.84	4.1
Pharmacy services	1 271 402.8	7.1
Auxiliary examinations	257 315.52	1.5
Public health services	8 819 343.5	51.3
Other services^a^	1 561 454.3	10.0
Total	17 175 997	100

^a^These services cannot be included in any category above. Administrative and other duties (report writing, continued professional education sessions and meetings, etc.) allocated to each category of community health professionals were included as well. Group interviews suggested their workload could account for 10% of the total

### Supply calculations

The current number of healthcare workers in post was 1752, while the annual supply of a full-time professional is 6000 EVs. The supply available in the 14 CHCs of Xicheng District is therefore approximately 10 512 000 EVs (Table 3).

**Table 3:** Demand, supply, gap, and surplus in CHS provision in Xicheng District, Beijing

	No. of community health professionals on the job (*a*)	Total demand in EV for CHS (*b*)	Total supply in EV for CHS (*c* = *a* × 6000)	Gap/surplus in EV (*d* = *c* − *b*)	No. of additional professionals required (6000 EVs per year per FTE employee) (*d*/6000)	Ratio (*e* = *c*/*b*)
Basic medical services	695	4 556 299.23	4 170 000	−386 299	64	0.92
Nursing services	519	3 355 984.89	3 114 000	−241 985	40	0.93
Public health services	276	6 173 540.45	1 656 000	−4 517 540	753	0.27
Other services^a^	262	1 528 718.36	1 572 000	43 282	7 (surplus)	1.03
Total	1752	15 614 543	10 512 000	−5 102 543	850	0.67

^a^Others include pharmacy services, auxiliary examination services, and services that cannot be included

### Calculation of “gap/surplus”

Table 3 illustrates the demand and supply calculations for each category of community health professionals and the gap or surplus for each. The ratio of supply to demand in the final column shows the magnitude of the shortage or surplus. For example, the total demand for basic medical services was 4 556 299.23 EVs. However, 695 doctors can only supply 4 170 000 EVs, so the gap is 386 299 EVs. To staff the gap, an extra 64 doctors is needed, giving a ratio of 0.92. As Table 3 shows, the total demand for CHS was greater than could be supplied for doctors, nurses, and public health workers. Providing the study population with the required standard of health services would need an additional 64 GPs, 40 nurses, and 753 public health workers. The category of other professionals, such as laboratorians and pharmacists, appears to be slightly over-staffed, with a ratio of supply to demand of about 1.03.

## Discussion

We used an economic-research-based approach to explore the capacity of community health professionals to meet demand for services in 14 CHCs in Xicheng District, Beijing. We created lists of CHS and estimated the person-time (equivalent value) for each service unit, based on a multi-stage iterative feedback and revision process. The total demand for CHS was calculated from the actual volume of CHS used by the residents of the sample areas in 2013. The total supply for CHS was calculated using staffing data supplied by the sample CHCs.

The study shows that the total supply and demand ratio for community health professionals in Xicheng District was 0.67, which suggests a significant unmet demand for services or overworking of human resources in CHS. This may be attributed to the greatly improved use of essential medical services and China’s national equalization of basic public health services in 2009. If the government of Xicheng intends to meet resident demand for CHS, and enable it to act as the gatekeeper of the health system, it will need to deliver significant improvements in CHS-staffing levels. Family doctors and nurses are in slight shortage, with a ratio of 92 and 93%, respectively. A 10% work pressure is always allowed, and therefore, it means the ratio for family doctor and nurses is fine as long as it is evenly distributed. Redistribution of current family doctor and nurses is recommended if the ratio varies across facilities. The current numbers of public health workers are a particular issue, being only 27% of the staff required to meet demand. In practice, because of the shortage of public health workers, 30% of nurses’ working time was estimated by the professionals involved to be allocated to the provision of public health services. Generally, one doctor potentially will generate more nursing work than one nurse can handle. It is particularly more significant that the doctor/nurse ratio be nearly 1:1 given that the nurses are providing public health services in addition to nursing care. This has led to concerns about the quality and deliverability of the NEPHSP and the nursing care as well. The calculation was based on the existing volume of service demand, quality service reaching the standard requirements, and no working overtime of professionals. Thus, the approach based on using existing levels of demand found a provider-shortage demand. The extra providers needed to improve the current poor quality and solve the problem of professionals working overtime which constituted the shortage of community health professionals.

Although the results of this study were obtained based on the actual situation in Xicheng District, they are also likely to reflect wider supply and demand imbalances among China’s urban community health professionals. Governments and relevant departments at all levels should therefore pay close attention to this issue. The nationwide implementation of the NEPHSP was designed to address increasing public health problems and improve the equity of China’s health service [11]. However, it did greatly increase the workload of primary healthcare facilities [34]. Effort is now needed to increase the numbers of public health workers in urban community health facilities and village hospitals in rural areas. It seems likely that use of CHS will increase over the next few years, with a series of supportive policies issued [6, 35, 36], and that the NEPHSP will expand to include more services and cover larger populations [29]. Additionally, the calculation was built on the assumption that the present way of delivering care was the most efficient way of delivering care and the existing levels of provider productivity are acceptable. With the improvement of the service delivery model and service productivity, the demand of community health professionals will change. This risks the development of further imbalances in supply and demand. A dynamic community health professional adjustment mechanism should therefore be established. Using this, the demand and supply of community health professionals, and the size of any gap or surplus, could be tracked in real time to enable timely action to address the problem.

This study did have some limitations. First, the approach primarily measures effort, but neglects issues of efficiency and effectiveness. More attention will be directed at the efficiency and effectiveness of various workers and combinations of workers providing the same service. Second, this study was based only in one location, a central urban region with a high population density. The results might be different in regions with different characteristics, such as low population density, or rural areas, especially those with mountains or forests. Future studies should examine different types of area. To our knowledge, this is the first time that the approach has been used to study the healthcare workforce in Chinese primary healthcare settings, and we suggest that the time standard and EV for each type of CHS proposed in this study could be applied to other similar districts in China and elsewhere.

## Conclusions

This study used an economic-research-based approach to calculate the number of community health professionals required to provide CHS in the Xicheng District of Beijing and then assessed the current staffing levels against this ideal. Our calculations indicated that significant numbers of new health professionals are required to deliver CHS. The current numbers of public health workers are a particular issue, being only 27% of the staff required to meet demand. A dynamic community health professional adjustment mechanism should be established with the increasing use of CHS and the improvement of service delivery model and service productivity.
